# Hidden Agenda - The Involvement of Endoplasmic Reticulum Stress and Unfolded Protein Response in Inflammation-Induced Muscle Wasting

**DOI:** 10.3389/fimmu.2022.878755

**Published:** 2022-05-09

**Authors:** Melanie Kny, Jens Fielitz

**Affiliations:** ^1^ Experimental and Clinical Research Center (ECRC), Charité-Universitätsmedizin Berlin, Max Delbrück Center (MDC) for Molecular Medicine in the Helmholtz Association, Berlin, Germany; ^2^ Department of Molecular Cardiology, DZHK (German Center for Cardiovascular Research), Partner Site, Greifswald, Germany; ^3^ Department of Internal Medicine B, Cardiology, University Medicine Greifswald, Greifswald, Germany

**Keywords:** endoplasmic reticulum stress, inflammation, intensive care unit acquired weakness, unfolded protein response, sepsis

## Abstract

Critically ill patients at the intensive care unit (ICU) often develop a generalized weakness, called ICU-acquired weakness (ICUAW). A major contributor to ICUAW is muscle atrophy, a loss of skeletal muscle mass and function. Skeletal muscle assures almost all of the vital functions of our body. It adapts rapidly in response to physiological as well as pathological stress, such as inactivity, immobilization, and inflammation. In response to a reduced workload or inflammation muscle atrophy develops. Recent work suggests that adaptive or maladaptive processes in the endoplasmic reticulum (ER), also known as sarcoplasmic reticulum, contributes to this process. In muscle cells, the ER is a highly specialized cellular organelle that assures calcium homeostasis and therefore muscle contraction. The ER also assures correct folding of proteins that are secreted or localized to the cell membrane. Protein folding is a highly error prone process and accumulation of misfolded or unfolded proteins can cause ER stress, which is counteracted by the activation of a signaling network known as the unfolded protein response (UPR). Three ER membrane residing molecules, protein kinase R-like endoplasmic reticulum kinase (PERK), inositol requiring protein 1a (IRE1a), and activating transcription factor 6 (ATF6) initiate the UPR. The UPR aims to restore ER homeostasis by reducing overall protein synthesis and increasing gene expression of various ER chaperone proteins. If ER stress persists or cannot be resolved cell death pathways are activated. Although, ER stress-induced UPR pathways are known to be important for regulation of skeletal muscle mass and function as well as for inflammation and immune response its function in ICUAW is still elusive. Given recent advances in the development of ER stress modifying molecules for neurodegenerative diseases and cancer, it is important to know whether or not therapeutic interventions in ER stress pathways have favorable effects and these compounds can be used to prevent or treat ICUAW. In this review, we focus on the role of ER stress-induced UPR in skeletal muscle during critical illness and in response to predisposing risk factors such as immobilization, starvation and inflammation as well as ICUAW treatment to foster research for this devastating clinical problem.

## Introduction

Skeletal muscle is one of the biggest organs in our body and of utmost importance for human health. It assures our vital body functions, such as breathing, protects our inner organs and maintains posture. Skeletal muscle serves as protein reservoir, which during consuming diseases assures survival. As a highly plastic organ skeletal muscle adapts to varying loading conditions, where increased load (e.g., training) leads to muscle growth and reduced load (e.g., immobilization) cause a decrease in muscle mass and muscle function, referred to as muscle atrophy. One of the most severe forms of muscle atrophy occurs in critically ill patients who often experience a diffuse symmetric weakness of all extremities and the diaphragm that is accompanied by muscle wasting, called intensive care unit-acquired weakness (ICUAW). ICUAW prolongs hospital-stay, prolongs mechanical ventilation and increases mortality. Some risk factors for ICUAW such as disease severity, inflammation and sepsis, insulin resistance, feeding status, and long-lasting mechanical ventilation have been identified but the molecular mechanisms are not well defined. However, a disturbed protein homeostasis with an increased protein degradation and a decreased protein synthesis has been implicated. Protein synthesis in myocytes is stringently controlled in the endoplasmic reticulum (ER, also called sarcoplasmic reticulum in myocytes) that also assures contractile calcium handling. The ER coordinates the synthesis, folding and posttranslational modification of membrane residing or secreted proteins. One third of newly synthesized proteins are defective, un- or misfolded. Also, physiological requirements and pathological conditions such as inflammation, viral infection, and hypoxia, may result in accumulation of defective proteins in the ER. Importantly, accumulation of damaged or unfolded proteins threatens ER function and is called ER stress. To resolve ER stress the Unfolded Protein Response (UPR) that aims to restore protein homeostasis by decreasing general protein synthesis, enhancing ER folding capacity, and mediating the degradation of unfolded proteins is initiated. If the UPR cannot resolve ER stress programmed cell death is induced. The UPR was reported to regulate muscle mass, inflammation, insulin resistance and starvation. Although these conditions are also implicated in ICUAW a direct link between ER stress-induced UPR and ICUAW has not been drawn.

**Figure 1:**
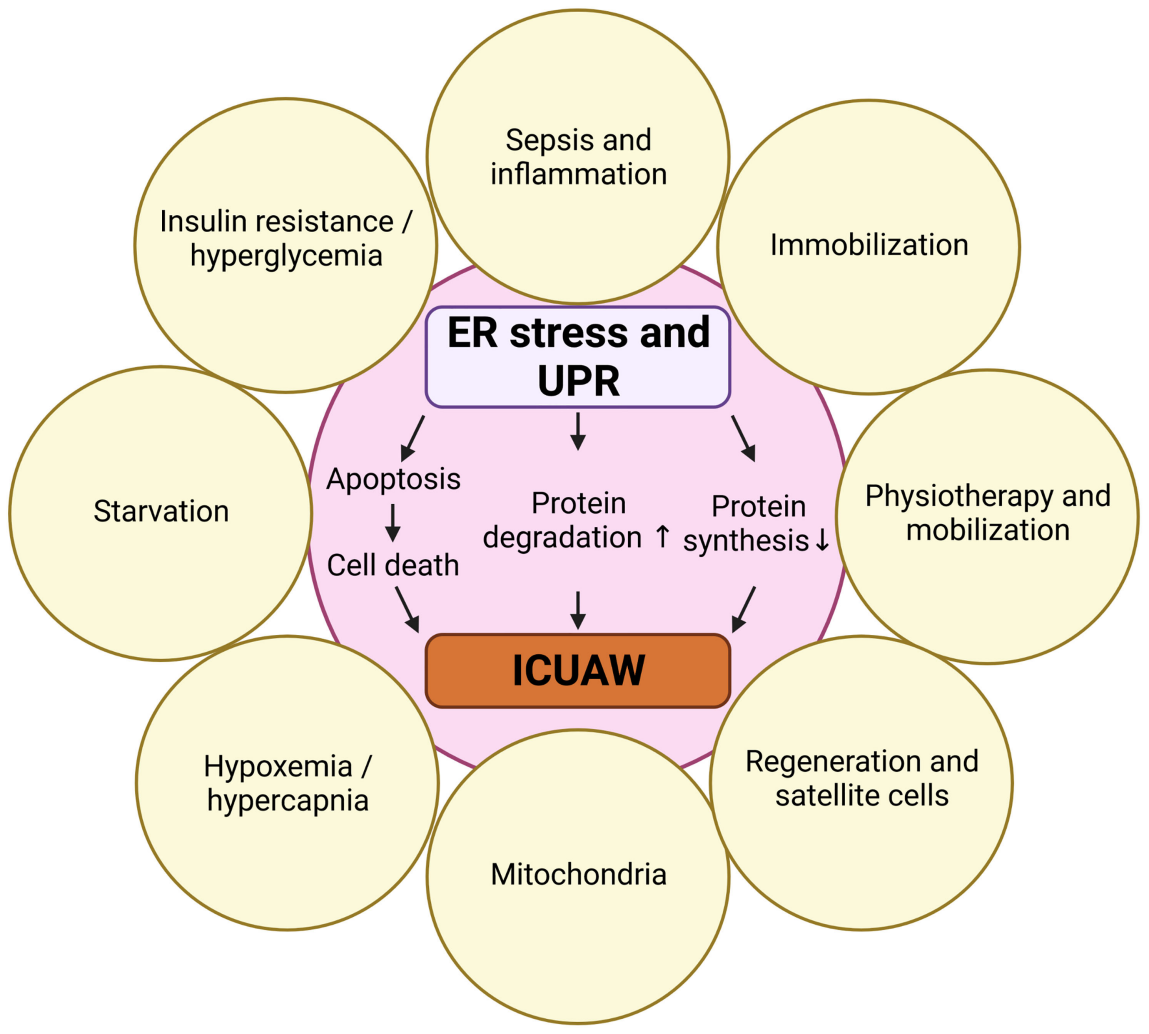
Risk factors for ICUAW that are also involved in ER stress and UPR. Created with BioRender.com.

Here we review the role of ER stress-induced UPR in skeletal muscle during critical illness and in response to predisposing risk factors. We first describe mechanisms of skeletal muscle atrophy and elute on the clinical syndrome of ICUAW. We then give an overview of protein quality control, ER stress and the different branches of UPR signaling. Recently, several ICUAW risk factors have been identified, and therefore we discuss how starvation, insulin resistance, immobilization, inflammation and sepsis, regeneration as well as physiotherapy and mobilization are interrelated with ER stress-induced UPR in muscle (**Figure 1**). Finally, we review the effects of UPR modulating compounds and chaperons on the UPR and discuss their potential to prevent or treat ICUAW.

## Mechanisms of Skeletal Muscle Atrophy

The skeletal muscle is one of the biggest organs in the human body and contributes approximately 40% of our body weight. It serves as a vital part in the musculoskeletal system and assures a multitude of functions, such as breathing, movement, maintaining body posture, protection of joints and inner organs as well as chewing and swallowing. It also serves as a huge protein reservoir that is used during periods of starvation or in life-threatening situations such as critical illness. Although the main purpose of this is to support survival mechanisms a continued usage of muscle as an energy source will eventually compromise its function. Because muscle contraction and movement are energy demanding skeletal muscle is a tissue that is metabolically active. Muscle uses glucose as a main energy source and any dysfunction in glucose uptake, such as occurring in insulin-resistance, will not only compromise muscle function but also has systemic effects (e.g., hyperglycemia).

Skeletal muscle mass is highly dynamic and is regulated by physiological and pathological stress such as exercise, nutrition, postnatal and adolescent growth, aging, cancer and inflammation. The response of the skeletal muscle to these stresses is an increase or a decrease in muscle mass and function. Deliberately, we here focus on circumstances that will reduce muscle mass. A physiological reduction in muscle mass (i.e., physiological muscle atrophy) mainly occurs due to unloading, such as in athletes with discontinued training, people with little physical activity, those having a sedentary life style or astronauts during space flight with a reduced gravitational load. Pathological muscle atrophy is caused by fasting, loss of innervation, due to neurological diseases (e.g., stroke, spinal cord or nerve injury), and accompanies various diseases, such as cancer (1), end-stage heart disease (2), end-stage renal disease (3), chronic obstructive pulmonary disease [COPD; (4)] or sepsis (5). A reduction in muscle mass and function has severe consequences for affected patients and its prevention is of utmost importance to human health. In general, protein homeostasis (i.e., proteostasis) in muscle is assured by a critical balance of protein synthesis, quality control and degradation. During muscle atrophy contractile proteins are degraded by the ubiquitin proteasome system (UPS), the autophagy-lysosomal pathway (ALP), and proteases such as calpains and caspases (6). Briefly, UPS-mediated protein degradation is a sequential process that leads to the degradation of target proteins by a large multiprotease complex called the proteasome. ALP works *via* the self-destruction of target containing lysosomes and mediates degradation of bulks of proteins and whole organelles. Calcium-dependent papain-like proteinases (Calpains) are lysosome independent cysteine proteinases and Caspases are Cysteine-Aspartate proteinases involved in programmed cell death. The function of the UPS and the ALP in physiological and pathological muscle atrophy are well understood. Whereas the ALP employs lysosomal proteolytic enzymes to degrade protein aggregates and membrane proteins, the UPS is a cytosolic and nuclear machinery that mediates targeted degradation of cytosolic and nuclear proteins. As part of protein quality control, the UPS is responsible for the degradation of defective proteins, such as incomplete, misfolded, denatured or oxidized proteins which tend to accumulate and form cytotoxic aggregates. Recently we have eluted on the role and regulation of UPS-dependent protein degradation in ICUAW [for review (6)]. Briefly, UPS-dependent protein degradation is key for many cellular processes, such as cell cycle control, development, signal transduction, and many more. The UPS is implicated in many diseases, such as cancer, neurological disorders or inflammation (7). During a process called ubiquitination, the 7.6 kDa polypeptide Ubiquitin (Ub) that is ubiquitously expressed (8, 9), is conjugated to the target protein resulting in mono- or polyubiquitination (8). This process requires the sequential action of three classes of enzymes: E1 (ubiquitin-activating), E2 (ubiquitin-conjugating) and E3 (ubiquitin-protein ligating) enzymes (10). In this cascade, the E1 ubiquitin-activating enzyme activates ubiquitin in an ATP-dependent manner facilitating the formation of a thioester and transferring the so modified ubiquitin to an E2 ubiquitin-conjugating enzyme. The E2 enzyme than interacts with its cognate E3 ubiquitin ligase to transfer the ubiquitin covalently to a target protein *via* an isopeptide bond. This cascade is repeatedly executed to generate ubiquitin chains of a minimum of four ubiquitin moieties on target proteins. The ubiquitin moieties can be linked at different lysine-residues of the ubiquitin amino acid sequence. For protein degradation lysine 48 (K48) linked poly-ubiquitin chains are most important as they are eventually recognized by the 26S proteasome were the so modified proteins are degraded (11). The specificity of the reaction is assured by E3 ubiquitin ligases that are also the rate limiting step. E3 ubiquitin ligases often display a tissue specific expression and a distinct subcellular distribution (12), which is thought to facilitate binding to their clients. Several E3 ubiquitin ligases have been described to play a role in skeletal muscle atrophy such as Muscle RING (really interesting new gene) finger 1 (MuRF1 encoded by Trim63), atrogin-1/MAFbx (encoded by Fbxo32), Muscle ubiquitin ligase of the SCF Complex in atrophy-1 (MUSA1, encoded by Fbxo30), Neural precursor cell expressed developmentally down-regulated protein 4 (NEDD4), and TNF receptor-associated factor 6 (TRAF6) (13–16). E3 ubiquitin ligases that are involved in muscle atrophy are often upregulated in muscle wasting conditions, and are therefore called atrogenes (13). They also assure substrate specificity for proteins to be degraded. For example, MuRF1 has been shown to associate with, ubiquitinate and mediate UPS-dependent degradation of the contractile proteins myosin heavy chain (MyHC) 2 and 4 (17). MuRF1 has also been shown to mediate the degradation of other structural proteins, such as alpha-actin, troponin I, troponin-T, telethonin, titin, nebulin, the nebulin-related protein NRAP, myosin light chain 2, myotilin and T-cap (18–21), as well as proteins that are involved in muscular energy metabolism such as muscle-type creatine kinase and glucocorticoid modulatory element binding protein-1 (22,23). Recent work suggests that MuRF1 functions as a component of a Cullin-type ubiquitin ligase encompassing Cullin 4A (Cul4A), DDB1, Rbx1 and DCAF8 to mediate ubiquitination and degradation of MyHC (24). Mice deficient for MuRF1/Trim63 and atrogin-1/Fbxo32 are protected against various forms of muscle atrophy (13) and pharmacological inhibition of MuRF1 has been shown to attenuate muscle atrophy (25,26). Previously we showed that MuRF1/Trim63 and atrogin-1/Fbxo32 mRNA and protein expression are increased in the vastus lateralis of critically ill human patients, especially those that were at risk to develop muscle wasting (27). Both E3 ligases were also upregulated in the tibialis anterior and gastrocnemius and plantaris of mouse models of neurogenic, inflammation- and starvation induced muscle atrophy (28–32), where their increased expression was associated with the atrophy phenotype and the loss of MyHC. We and others recently summarized the current knowledge about factors and signaling pathways involved in the regulation of E3 ubiquitin ligases relevant for muscle atrophy (6,33,34). For this review it is relevant to know that proinflammatory cytokines (e.g., Tumor Necrosis Factor (TNF), Interleukin 6 (IL-6), IL-1β, TNF-Related Weak Inducer of Apoptosis (TWEAK), members of the Transforming Growth Factor (TGFs) family) and acute phase proteins [e.g., Serum Amyloid A1 (SAA1)] are well known factors involved in muscle wasting (6,33,34). To cause muscle atrophy, these cytokines bind to their receptors at the myocyte cell surface and activate transcription factors and signaling proteins such as Nuclear Factor-κB (NF-κB), JAK/STAT, SMAD, MAPK, TFEB/TFE3, and IGF/PI3K/AKT to regulate MuRF1/Trim63 and atrogin-1/Fbxo32 expression that in turn mediate muscle atrophy (6,33,34). Importantly, in addition to the immune system the skeletal muscle itself contributes to the production and release of acute phase response proteins and proinflammatory cytokines (28,31,32,35), which may aggravate muscle loss especially during chronic inflammation and sepsis. Although we start to understand the role of protein degradation and its regulation in muscle atrophy only few therapeutic concepts arose from these insights.

### Intensive Care Unit-Acquired Weakness

Generalized weakness that is accompanied by muscle wasting is a dominant clinical problem for many critically ill patients during treatment at the intensive care unit (ICU) but also after ICU discharge. This syndrome is called intensive care unit (ICU)-acquired weakness (ICUAW), which is defined as clinically detected weakness in critically ill patients where the only plausible etiology is the critical illness itself (36). To rule out alternative causes of weakness, it is requested that the manifestation of ICUAW must follow the commencement of critical illness. About 40% of critically ill patients experience ICUAW. A major contributor to ICUAW is a reduced muscle mass and function. Indeed, during the first 10 days in ICU critically ill patients lose around 20% of muscle mass (37). Due to the nature of muscle function tests that require cooperative patients, in most patients the decreased muscle function is not diagnosed until after the acute disease phase, e.g., when the patients awake from sedation (38). Therapeutic interventions at this stage are tedious and require lots of resources. An early detection of ICUAW and a risk factor-based treatment could be more effective, and such clinical studies have been performed (39–41). Patients with ICUAW show a pronounced disparity between movement and their level of consciousness and cooperativity. Physical examination often reveals diffuse, symmetric weakness of all extremities and the diaphragm. The decreased muscle function is associated with a prolonged ICU- and hospital-stay, and an increased mortality. Additionally, weakness of the diaphragm and the auxiliary respiratory muscles, prolongs mechanical ventilation and hampers ventilator weaning (42–44). Persisting weakness is associated with functional limitations, lower employment rates and a diminished quality of life (45,46). ICUAW patients often encounter limitations even in simple daily activities such as getting out of bed, getting out of a chair and going up and down stairs. Sequelae of ICUAW may persist for years after ICU- or hospital-discharge, which is in contrast to physiological muscle atrophy, which is reversible and responds well to physiotherapy and exercise. The pathophysiology of ICUAW is still elusive. A recent meta-analysis revealed that disease severity (i.e., Acute Physiology and Chronic Health Evaluation II score), sepsis, multiple organ failure, hyperglycemia (i.e., insulin resistance), electrolyte disturbances, parenteral nutrition (i.e., feeding status), female sex, medication (e.g., neuromuscular blocking agents, aminoglycoside antibiotics and norepinephrine) and long-lasting mechanical ventilation are risk factors for ICUAW (47). Because 60 to 100% of patients with sepsis develop ICUAW, this risk factor is of utmost importance. Sepsis, which is defined as a life-threatening organ dysfunction caused by a dysregulated host response to an infection (48,49), is the predominant cause of ICU-admission and the prevailing cause of death in ICU (50). Almost 30% of critically ill patients are either admitted with or become septic during ICU treatment (51, 52). Since systemic inflammation in sepsis is strongly associated with muscle atrophy and ICUAW in nearly all patients, a better understanding of the underlying pathomechanisms is required to prevent or treat the disease.

The reduction in muscle mass of critically ill patients is associated with a decreased myofiber cross-sectional area (MCSA). In general, skeletal muscle is composed of myofibers with different contractile behaviors, so called type I/slow- and type II/fast-twitch myofibers. The contractile behavior of myofibers depends on the myosin heavy chain (MyHC) that they predominantly express and based on these differences fast and slow contracting muscles are differentiated. For example, the soleus muscle predominantly contains type I/slow contracting MyHC-I, encoded by MYH7 and is therefore considered a slow muscle, whereas the tibialis anterior muscle mainly contains type II/fast contracting MyHC-IIA, MyHC-IIB or MyHC-IIx, encoded by MYH2, MYH4 and MYH1, respectively, and is therefore referred to as a fast muscle. Importantly, the degree of slow/type I- and fast/type II-fiber atrophy is different. Specifically, although histological analyses of biopsy specimens from the vastus lateralis muscle of ICU patients revealed that the MCSA of all fiber types is reduced myofiber atrophy is much more pronounced in fast twitch/type II-myofibers as opposed to slow twitch/type I-myofibers. This is associated with a reduction in fast and slow myosin heavy chain (MyHC) protein and mRNA expression (27). The consequences of myofiber atrophy are a decrease in muscle function with reduced specific force and endurance (53). In this review we will focus on the importance of the ICUAW-risk factors inflammation and sepsis, insulin resistance, feeding status and immobilization as well as muscle activation for the disease course.

## Protein Quality Control, Endoplasmic Reticulum Stress and the Unfolded Protein Response

Like protein degradation also protein synthesis is stringently controlled in myocytes. These control mechanisms occur in the endoplasmic reticulum (ER) that in muscle is also called sarcoplasmic reticulum. The ER is a cytoplasmatic membrane network consisting of tubules, vesicles and cavities that reaches from the nuclear membrane into the cytoplasm. In myocytes, the ER is highly specialized for contractile calcium handling. Importantly, the ER also coordinates the synthesis, folding, posttranslational modification and maturation of proteins, which is particularly relevant for secreted and membrane residing proteins. Soluble proteins that are meant to be processed in the ER harbor an N-terminal signal peptide, which is often cut upon import into the ER (54). The recognition of the signal peptide occurs either during translation (co-translational protein import) inducing a translational arrest until the ribosome has docked to the ER or after translation is completed, which requires cytosolic chaperones (e.g., heat shock protein 70 and 40 family members) that hold the proteins in an import competent unfolded state (55, 56). The oxidizing and calcium containing ER lumen provides an environment that enables protein modification and maturation, which is virtually impossible in the cytoplasm, e.g., disulfide bond formation, glycophosphatidylinositol (GPI)-anchor addition, N-linked glycosylation, protein folding and chaperone functionality (57). Within the process of translation, translocation and processing, nascent proteins are constantly monitored by a complex system of ER chaperones assuring assistance for posttranslational modification as well as folding and quality control. Among these chaperones are heat shock proteins, such as glucose regulated protein 78 (GRP78, also referred to as binding immunoglobulin protein (BiP) or HSPA5), lectins, such as Calnexin or Calreticulin, or thiol-disulfide oxidoreductases such as protein disulfide isomerase (PDI) (58, 59). Despite this well-regulated quality control system, around 30% of newly synthesized proteins are defective, un- or misfolded (60). Moreover, mature proteins can be damaged by environmental or pathophysiological conditions. Also, the protein folding capacity of the ER can be disturbed by a perturbed ER calcium homeostasis, hypoxia, viral infection, increased protein cargo or altered protein glycosylation. These conditions can lead to the accumulation of defective proteins in the ER, which is toxic to the cell and threaten cell viability, and is called ER stress. To counteract and resolve accumulation of damaged or unfolded proteins, ER stress induces signaling pathways, which are altogether named the Unfolded Protein Response (UPR). The UPR causes a decrease in general protein synthesis, enhances the ER folding capacity, and mediates the degradation of unfolded proteins. If the UPR is not sufficient to resolve ER stress, Janus Kinases (JNK) and Caspases 3, 7 and 12 are activated to induce programmed cell death (61, 62).

**Figure 2:**
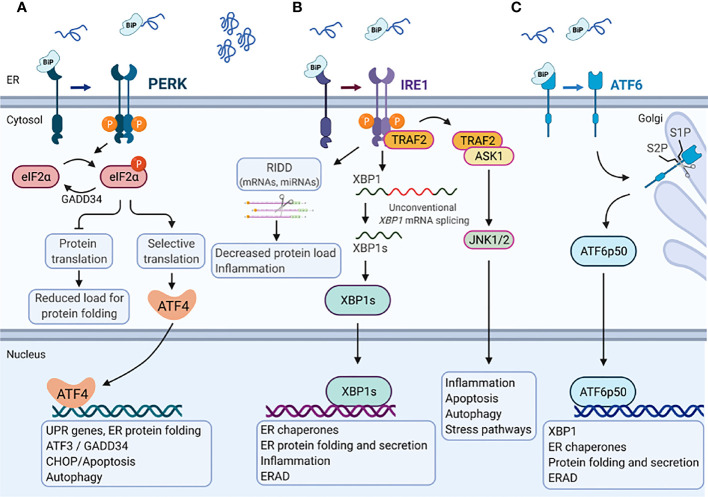
Endoplasmic reticulum stress-mediated Unfolded protein response. In the absence of ER stress, the ER chaperone protein GRP78/BiP is bound to the three major branches of the unfolded protein response (UPR) **(A)** PERK, **(B)** IRE1, and **(C)** ATF6 and keeps them in an inactive state. Upon accumulation of misfolded or unfolded proteins that cause Endoplasmic reticulum (ER) stress, GRP78/BiP dissociates from these receptors and binds to misfolded proteins, which activates the UPR signaling proteins. **(A)** PERK phosphorylates eukaryotic translation initiation factor 2 subunit-α (eIF2α), decreasing mRNA translation. However, specific mRNAs (e.g., ATF4) mRNA can be translated in the presence of phosphorylated eIF2α. ATF4 activates the transcription of UPR target genes that encode proteins involved in ER protein folding, autophagy and apoptosis (e.g., CCAAT/enhancer- binding protein homologous protein (CHOP) and GADD34). GADD34 targets protein phosphatase 1 (PP1) to dephosphorylate eIF2α which reactivates mRNA translation. **(B)** IRE1α has endoribonuclease activity and splices XBP1 mRNA to XBP1s, which encodes a transcription factor that activates expression of UPR target genes such as chaperones, ER folding and secretion, inflammation and ERAD. Activation of the IRE1α endoribonuclease activity can also induce the degradation of mRNAs encoding membrane or secreted proteins by regulated IRE1-dependent decay (RIDD), which affects the protein folding load, inflammation and inflammasome signaling. The cytosolic domain of IRE1α is also able to interact with tumor necrosis factor receptor-associated factor 2 (TRAF2), which can activate ASK1 and JNK1/2 to mediate inflammation, apoptosis, autophagy and stress pathways. **(C)** ATF6 moves from the ER to the Golgi apparatus, where it is cleaved by site-1 protease (S1P) and site-2 protease (S2P). The cleaved product (ATF6p50) migrates to the nucleus and activates the transcription of XBP1 as well as genes that are involved in ER protein folding and secretion, encode ER chaperones, and ERAD components. Created with BioRender.com.

The UPR is mediated by three ER transmembrane UPR-signal transducers, the inositol-requiring protein 1 (IRE1), the protein kinase R (PKR)-like endoplasmic reticulum kinase (PERK) and the cAMP-dependent transcription factor 6 (ATF6) (63). Roughly, in the absence of ER stress, the ER residing chaperone protein GRP78/BiP is bound to PERK, IRE1 and ATF6, and keeps them in an inactive state. Upon ER stress, GRP78 dissociates from these receptors and binds to misfolded proteins, which activates the UPR signaling proteins (**Figure 2**) (64).

IRE1, a type I transmembrane protein, is conserved from yeast to human with two paralogues in Mammalia, IRE1α and IRE1β. Their cytosolic domain comprises a serine-threonine kinase and an endoribonuclease activity. In response to ER stress GRP78 dissociates from IRE1, which in turn dimer- or oligomerize, become autophosphorylated and activates its own ribonuclease activity. IRE1 activation results in spliceosome-independent splicing of a 26-base intron from X-box-binding protein 1 (XBP1) mRNA (65). Spliced XBP1 (XBP1s) acts as a transcription factor that increases the expression of genes encoding ER chaperones, transcription factors (e.g., XBP1) and components of the secretory pathway as well as proteins involved in ER-associated degradation (ERAD) (66). Activation of IRE1 also induces rapid turnover of mRNAs encoding membrane or secreted proteins in a process called regulated IRE1-dependent decay (RIDD) (67). However, long lasting ER stress causes binding of IRE1α to TNF receptor-associated factor 2 (TRAF2), which in turn recruit’s apoptosis signal-regulating kinase 1 (ASK1) and activates the apoptotic c-Jun amino-terminal kinase (JNK) as well as the p38 pathway to initiate cell death (68, 69).

PERK, a type I transmembrane protein, binds GRP78 in its inactive state and has a cytosolic domain with kinase activity. In response to ER stress, PERK undergoes autophosphorylation and oligomerization. Activated PERK phosphorylates eukaryotic translation initiation factor 2α (eIF2-α) on serine 51, resulting in general inhibition of translation. However, eIF2-α also increases the translation of some mRNAs, such as that of the transcription factor activating transcription factor-4 (ATF4), which in turn induces UPR target genes (70). ATF4 increases the expression of the transcription factors ATF3 and C/EBP homologous protein (CHOP). ATF3 together with ATF4 promote the expression of growth arrest and DNA damage-inducible protein (GADD34), a regulatory subunit of protein phosphatase 1 (PP1), leading to dephosphorylation of eIF2-α which re-activates protein synthesis (62). In contrast, CHOP induces multiple pro-apoptotic molecules and is therefore considered a pro-apoptotic protein (71).

ATF6 is a type II transmembrane protein, that in response to ER stress moves from the ER to the Golgi apparatus where it is cleaved by Site-1 protease and Site-2 protease. This process is called regulated intermembrane proteolysis. The cleaved N-terminal fragment of ATF6 is a basic leucine zipper (bZIP) transcription factor that harbors DNA binding and transcription activation domains (72, 73). Upon cleavage ATF6 translocates to the nucleus where it cooperates with XBP1s to bind to ER stress response elements (ERSE) in the promoter region of genes encoding proteins that counteract ER stress, such as GRP78, GRP94 and Calnexin to induce their expression. The activation and downstream signaling pathways of the different UPR branches have been reviewed in further detail recently (63).

For removal of misfolded proteins from the ER, the quality control system of the ER sorts them for ER-associated protein degradation (ERAD) *via* the proteasome. The UPR activates the transcription of ERAD genes (74). The process of ERAD follows four consecutive steps: misfolded proteins are 1) recognized by GRP78/BiP and transferred to the ER membrane where they are 2) ubiquitinated and retrotranslocated by E3 ubiquitin ligase containing multiprotein complexes and are 3) extracted from the ER membrane by the p97 complex, and 4) are finally transferred to the 26S proteasome for degradation [reviewed in (75)].

The critical importance of the UPR for cell survival and organ function is supported by genetic studies. For example, homozygous Grp78/BiP knockout mice show pre-implantation lethality by embryonic day 3.5, with greatly reduced proliferation of embryonic cells and increased apoptotic death of the inner cell mass that are the precursors of embryonic stem cells (76). These data indicate that GRP78 is indispensable for embryonic cell growth and pluripotent cell survival. In contrast, heterozygous Grp78/BiP mice, in which the GRP78/BiP levels are halved in adult tissues, are viable and phenotypically normal (76). In addition, although Perk1-knockout mice are morphologically normal at birth, they die postnatally due to β-cell degeneration in the pancreas resulting in diabetes mellitus, reduced exocrine function of the pancreas with attenuated secretion of digestive enzymes, and skeletal defects (77). Similarly, homozygous Eif2aS51A-mutant mice that express a phosphorylation resistant form of eIF2α die within 18 hours after birth due to hypoglycemia associated with defective gluconeogenesis (78). Both Ire1- and Xbp1-deficient mice are embryonically lethal due to defective B cell lymphopoiesis and liver failure, respectively (79). Single Atf6a and Atf6b-knockout mice develop normally, whereas Atf6a and Atf6b-double knockout mice are embryonically lethal (80). In addition, when exposed to ER stress-inducing reagent tunicamycin (an inhibitor of N-glycosylation in the ER) by intraperitoneal injection the Atf6a-knockout mice display liver dysfunction and steatosis (81). In summary, these data show that the UPR is particularly important for proliferative and secretory cells, which facilitated research especially in cells and organs and their pathologies were these aspects are important. In contrast, in skeletal myocytes and muscle associated pathologies these features have not been recognized as that important for a long time. Despite the fact that the skeletal muscle harbors a large network of ER, the function of ER stress and the UPR in the regulation of physiological skeletal muscle function and diseases is not well understood. However, current studies reveal that an activation of the UPR can have beneficial as well as deleterious effects for the skeletal muscle (82). In the following paragraphs, we review the available literature about the role of the UPR in the skeletal muscle focusing on ICUAW and associated pathologies.

### Risk Factors of ICUAW and Their Involvement in ER Stress-Induced UPR

#### Starvation-Induced ER Stress and ICUAW

Appropriate nutrition is often an issue in the care of critically ill patients especially during the first days in ICU that often lead to missed caloric goals. Adequate feeding and outcome are closely connected with each other. Both underfeeding and overfeeding is often observed in critically ill patients, which can be harmful to critically ill patients suggesting that caloric goals need to be correctly determined and reached (83). Underfeeding and overfeed are related to uncertainties about the energy expenditure (resting metabolic rate, additional requirements due to the illness that is also dynamic with the disease course), the underlying disease itself, unknown body composition, difficulties to administer food, and the route of enteral versus parenteral nutrition. Patients biological age and sex differences may also have an effect on energy expenditure. Uncertainties about the right composition of the administered calories complicate the situation. As we describe later in this review hyperglycemia and insulin resistance are risk factors for ICUAW. It is therefore reasonable to assume that treating these risk factors may improve clinical outcome. This hypothesis led to the clinical concept of permissive underfeeding, which has been shown to reduce blood glucose and insulin levels indicative for an improved insulin sensitivity (266). However, Arabi et al., investigated if permissive underfeeding has an effect on clinical outcome in critically ill patients. They showed that administration of a hypocaloric diet with 70% of calculated caloric requirements (permissive underfeeding) did not affect mortality, ICU stay and other secondary outcomes when compared to standard nutrition (266). The study design required that both groups received the same amount of proteins implicating that not the number of calories administered but that the amount of proteins could have an effect on outcome of critically ill patients. The problematic situation on feeding of critically ill patients has been reviewed elsewhere (267–270). Although experimental models suggest an association between the feeding status and UPR, its involvement in this complex situation is far from being understood.

Importantly, fasting is a strong stimulus for skeletal muscle atrophy (29), which shares common pathways with other causes of muscle wasting (84). The UPS and the ALP are the best described pathways involved in starvation-induced muscle atrophy. Recent work indicates that also the UPR is involved in this pathology (85). Specifically, 24 hours of starvation caused an elevation of mRNA and protein expression of UPR markers such as Atf4, Chop, Grp94, and Gadd34 and an increased splicing of XBP1 in the gastrocnemius muscle of mice. Also, 48 hours of starvation in cows leads to an increased IRE1α phosphorylation and an increased XBP1 splicing in hepatocytes, indicating a connection between ER stress and starvation (86).

Additionally, TNF Receptor Associated Factor 6 (TRAF6) an E3 ubiquitin ligase that belongs to a family of conserved intracellular adaptor proteins has been shown to be involved in UPR. In response to cytokines and microbial products, TRAF6 regulates multiple signaling pathways, such as NF-κB, MAPK, and PI3K/Akt. Skeletal muscle specific deletion of Traf6 attenuates skeletal muscle atrophy caused by starvation, denervation, and cancer cachexia (15, 85). Of note, TRAF6 is essential for the inducible expression of ER stress response-related genes in response to fasting. Specifically, the starvation-induced increase in mRNA and protein expression of the UPR markers ATF4, CHOP, GRP94, and GADD34 in the gastrocnemius muscle was attenuated in muscle specific Traf6 knockout mice (85). However, the mechanism by which TRAF6 and the UPR are connected in skeletal muscle during starvation remains to be uncovered.

The activating transcription factor 4 (ATF4) that is downstream of the PERK arm, was also shown to mediate starvation-induced muscle atrophy. Of note, 24 hours of fasting increased the Atf4 mRNA expression in tibialis anterior of male mice (87). Inhibition of ATF4 expression by electroporation of a small interfering RNA targeting ATF4 or overexpression of a phosphorylation-resistant form of eIF2α (eIF2α-S51A) attenuated myofiber atrophy in tibialis anterior muscle during fasting (87). Overexpression of ATF4 caused a reduction in myofiber size even in the absence of fasting. Importantly, electroporation of a transcriptionally inactive ATF4 construct (ATF4ΔbZIP) did not reduce myofiber size, suggesting that ATF4-dependent gene expression is required for ATF4-mediated atrophic effects (87). These data show that ATF4 mediates starvation-induced muscle atrophy but it can also cause muscle atrophy in the absence of starvation.

Starvation has also been shown to activate the nutrient-sensitive transcription factors transcription factor EB (TFEB) and transcription factor E3 (TFE3). Both TFEB and TFE3 belong to the microphthalmia/transcription factor E (MiT/TFE) family of basic bZIP transcription factors that recognize a unique E-box motif within the proximal promoters of lysosomal and autophagy genes (88, 89), and regulate cellular catabolism and nutrient-dependent lysosomal response (90, 91). Both TFEB (92) and TFE3 (89) are predominantly localized in the cytoplasm but also to the nucleus (93, 94). In the presence of nutrients, mTORC1 (mammalian target of rapamycin complex 1) phosphorylates TFEB and TFE3, which facilitate their binding to 14-3-3 chaperone proteins and mediates their retention in the cytoplasm. Conversely, during starvation reduced mTORC1 activity increases the shuttling of TFEB (89) and TFE3 (89) into the nucleus increasing the expression of TFEB- and TFE3-dependent genes. Importantly, ER stress also induces the nuclear translocation of TFEB and TFE3 and this effect was shown to be dependent on PERK and calcineurin but not mTORC1 (95). We recently described that TFEB (93) and TFE3 (94) are also localized to the nucleus where TFEB is bound to conserved E-box elements in the MuRF1/Trim63 promoter to regulate its expression (93) implicating a role in muscle atrophy. Both TFEB and TFE3 are inhibited by class IIa histone deacetylases (i.e., HDAC4, 5, and 7) and this inhibition is reversed by the Protein kinase D family (i.e., PKD1, 2 and 3). However, if this pathway is important in ER stress induced muscle atrophy has not been investigated.

#### Insulin Resistance, Hyperglycemia and UPR

Insulin resistance accompanied by hyperglycemia is common in critically ill patients and strongly associated with increased morbidity and mortality (96). The underlying mechanism is still elusive but includes a decreased activity at the level of the insulin receptor and the post-receptor level (97). The insulin receptor is a receptor tyrosine kinase and its activation by insulin causes the cytosolic domains to autophosphorylate. The activated insulin receptor phosphorylates tyrosine residues in proteins such as insulin receptor substrate 1 (IRS-1) and IRS-2 that mediate downstream signaling. IRS-1 activates PI3K and Akt, which regulate muscle protein homeostasis (98). In addition, activation of the insulin receptor *via* the Akt substrate of 160 kDa leads to translocation of the glucose transporter (GLUT)-4 into the plasma membrane, which increases cellular glucose uptake (99). Insulin resistance causes decreased glucose uptake in skeletal muscle. Under physiological conditions myocytes internalize circulating glucose *via* their main glucose transporter glucose transporter-4 (GLUT4). GLUT4 is contained in storage vesicles that are localized near the sarcolemma, the transverse tubular system and in the trans-Golgi region (100). Normally, these GLUT4 storage vesicles are translocated to the plasma membrane and glucose uptake is increased in skeletal muscle in response to insulin (101). In insulin resistance insulin receptor signaling is perturbed, GLUT4 translocation is attenuated and circulating glucose cannot be taken up. Since glucose is the main source of energy in muscle, less energy-rich phosphates (e.g., ATP) are formed, which reduces muscular energy supply (102). This impairs muscle function, especially strength and endurance. At the same time, insulin has an anabolic effect on muscle by increasing protein synthesis and inhibiting protein breakdown (98, 103), so that insulin resistance leads to disturbed protein homeostasis with increased protein breakdown and reduced protein synthesis, resulting in muscle atrophy and weakness (27, 37). Importantly, insulin resistance has been reported to be a predictor for ICUAW (47). Indeed, previously we reported that GLUT4 was trapped in the perinuclear space of myofibers of critically ill patients whereas it was localized to the sarcolemma of myofibers of control subjects (102). This observation was most pronounced in patients with critical illness myopathy, a severe form of ICUAW further supporting that insulin resistance is involved in ICUAW.

Although the contribution of the UPR to insulin resistance in skeletal muscles of the critically ill is uncertain, published evidence supports this assumption. For example, ER stress has been shown to be associated with insulin resistance, which was accompanied by a suppression of insulin receptor signaling and serine phosphorylation of insulin receptor substrate-1 (IRS-1), *in vivo* and *in vitro* (104). Insulin resistance was also described in Xbp1 knockout mice (104). Inhibition of ER stress by the chemical chaperones 4-phenyl butyric acid (4-PBA) or taurine-conjugated ursodeoxycholic acid (TUDCA) restored systemic insulin sensitivity, enhanced the action of insulin on muscle, and normalized hyperglycemia in obese and diabetic mice (105). These data indicate that inhibition of ER stress by the chemical chaperones 4-PBA and TUDCA counteracts insulin resistance.

The adenosine 5’-monophosphate (AMP)-activated protein kinase (AMPK) that senses and regulates cellular energy states increases the expression of GLUT4 (106). Deletion of Ampk2a (encoding AMPK2α) in mice causes a reduction in glucose utilization in skeletal muscle (107). Activation of ER stress by tunicamycin or thapsigargin (an inhibitor of ER-specific calcium ATPase) reduces AMPK activity, insulin signaling and glucose uptake in rat-derived L6 myotubes, mainly due to an increased extracellular signal-regulated kinase (ERK) phosphorylation. Inhibition of ERK restored AMPK phosphorylation and glucose uptake that were deteriorated in response to ER stress (108). These data implicate a cross talk between ER stress and the major energy sensor AMPK in myocytes.

Insulin resistance caused by the saturated fatty acid palmitic acid also caused ER stress as indicated by an increased mRNA and protein expression of the ER stress-markers ATF6, ATF3, GRP78, CHOP, and Tribbles 3 (TRIB3) in C2C12 myotubes. Palmitic acid-induced ER stress, insulin resistance and, inflammation were attenuated by low concentrations of the proteasome inhibitor Bortezomib and these effects were mediated by AMPK. Importantly, knockdown of AMPK abolished the protective effects of Bortezomib (109). These studies indicate that ER stress induced insulin resistance is at least partially mediated by inhibition of AMPK activity.

The pseudokinase Tribbles 3 (TRIB3) that is expressed in various tissues such as liver, adipose tissue, heart, and skeletal muscle (110) has also been shown to impede insulin signaling (111). TRIB3 is increased in skeletal muscle of patients with type 2 diabetes mellitus known to be insulin resistant (110). TRIB3 binds to Akt and inhibits Akt phosphorylation eventually preventing insulin-stimulated glucose uptake in C2C12 myocytes (112). Activation of ER stress by tunicamycin and thapsigargin causes an increase in TRIB3 in C2C12 cells and skeletal muscle, which possibly mediates ER stress-induced insulin resistance. Accordingly, when TRIB3 was overexpressed in tibialis anterior of mice insulin signaling was greatly impaired (113). In addition, deletion of Trib3 in mice prevented high-fat diet induced insulin resistance in skeletal muscle (113). If Tribbles 3 plays a role in insulin resistance in skeletal muscle of the critically ill is not known.

The Protein tyrosine phosphatase 1B (PTP1B) that is a key negative regulator of insulin signaling by dephosphorylation of tyrosine residues of the insulin receptor which inhibits insulin receptor signaling (114). PTP1B that is localized on the ER membrane has also been shown to mediate insulin resistance in response to ER stress (115). Specifically, high-fat diet (for 20-weeks) induced ER stress (e.g., GRP78) caused an increase in PTP1B expression in the gastrocnemius of mice. This response was attenuated by administration of TUDCA for 5-weeks. Likewise, tunicamycin-induced ER stress caused an increase in PTP1B levels in C2C12 myotubes. In addition, tunicamycin-induced phosphorylation of eIF2α and JNK2 were attenuated in Ptpn1 knockout mice, encoding PTP1B. Downregulation of PTP1B or inhibition of ER stress by TUDCA treatment improved glucose uptake in tunicamycin treated C2C12 myotubes (116). These data indicate that ER stress activates PTP1B and that PTP1B is required for activation of ER stress pathways to mediate insulin resistance in skeletal muscle. In addition, it has been reported that mice harboring a germline deletion of Ptpn1 were protected against high-fat diet induced insulin resistance, weight gain, and hepatic steatosis. These mice also exhibited improved glucose uptake and Akt phosphorylation in the gastrocnemius. In addition, high-fat diet-induced increase in GRP78/BiP and phosphorylation of eIF2α as well as JNK2 were significantly blunted in Ptpn1 knockout mice. These data support the hypothesis that ER stress induces the expression of PTP1B which mediates insulin resistance in the skeletal muscle in response to high-fat diet (117). It has also been shown that PTP1B potentiates IRE1-mediated ER stress signaling pathways in mouse embryonic fibroblasts (118).

Hyperglycemia leads to elevated glucosamine levels that are also able to induce insulin resistance and ER stress as indicated by an increased expression of the ER stress markers GRP78/BiP, XBP1 and ATF6 in both human and L6 myotubes (119). Pretreatment of both rat and human myotubes with 4-PBA or TUDCA, attenuated glucosamine-induced ER stress and insulin resistance. Glucosamine reduced the expression of GLUT4, myocyte enhancer factor 2A (MEF2A) and peroxisome proliferator-activated receptor gamma coactivator-1 alpha (PGC-1α) expression, which was prevented by 4-PBA and TUDCA in both rat and human myotubes. Importantly, glucosamine-mediated downregulation of GLUT4, MEF2A and PGC-1α was attenuated by ATF6 silencing. These data strongly indicate that the ATF6 arm of the UPR mediates glucosamine-induced insulin resistance (119).

In summary, these studies indicate that ER stress and UPR pathways are involved in systemic and muscular insulin resistance and muscular glucose uptake. However, if this pathomechanism is involved in ICUAW and if its targeting has therapeutic potential requires further investigation.

#### Immobilization-Induced Muscle Atrophy in the Critically Ill

Immobilization of critically ill patients will inevitably lead to disuse and unloading of skeletal muscle which are major risk factors for ICUAW (21). Although immobilization allows complex treatments in ICU, the accompanying unloading of the skeletal muscle will lead to skeletal muscle atrophy and a reduction in muscle force. The loss of muscle mass and function delays recovery and attempts to prevent muscle atrophy through physical therapy or other interventions have been insufficient. Despite the fact that skeletal muscle atrophy due to immobilization is a prevalent and severe clinical problem in many different medical situations, the underlying mechanisms are not well understood. Of note, bed rest in humans and immobilization of animals have been shown to cause skeletal muscle atrophy mainly by an increased activity of UPS-mediated protein degradation with enhanced expression of MuRF1 and atrogin1 (27, 29–32, 120). Since skeletal muscle atrophy due to disuse leads to an upregulation of genes encoding sarcoplasmic reticulum (SR) calcium-handling proteins and considering that many of the proteins that are induced with ER stress are also calcium-handling proteins a connection between disuse atrophy and ER stress is likely. This hypothesis was tested in soleus muscle of rats after seven days of unloading. In this model, the expression of the UPR markers GRP78/BiP, calreticulin, CHOP and eIF2α remained unchanged in response to inactivity (121). Similar observations have been made by Baehr et al., were hindlimb unloading did not cause ER stress in soleus of adult (9 month) and old (29 month) male rats (122). However, ER stress was different between soleus and tibialis anterior of the same animals and revealed age-specific effects. The authors also investigated if reloading of the muscle show age-specific effects on ER stress. Indeed, in response to 14 days of reloading, both GRP78/BiP and CHOP were increased in soleus of adult but not old mice. Also, CHOP increased upon reloading in tibialis anterior of old but not adult rats, whereas GRP78/BiP remained unchanged (122). Contrary to the mentioned data, an upregulation of ER stress and UPR pathway genes in response to immobilization was reported by other groups. For example, 10-days of bed rest resulted in a persistent upregulation of the UPR in skeletal muscle of young men, as shown by microarray analysis (123). In addition, the expression of ATF4, a downstream transcription factor of PERK branch, increases in response to 3 days of unilateral hindlimb immobilization in tibialis anterior in mice (124). Furthermore, conditional Atf4-knockout mice lacking ATF4 in muscle are resistant to immobilization-induced muscle atrophy, fasting-induced muscle atrophy, and age-related muscle atrophy (87, 124–126). Conversely, forced expression of ATF4 is sufficient to induce muscle atrophy even in the absence of inactivity (87, 126). Further studies that address the individual UPR pathways are needed to elucidate their specific role of ER stress in disuse-induced skeletal muscle atrophy.

Denervation is another model to investigate mechanisms of immobility-induced muscle atrophy. To achieve this phenotype the sciatic nerve is cut and dissected in rodents (29). It has been shown that denervation leads to an increased phosphorylation of eIF2α and splicing of XBP1 mRNA in the gastrocnemius 3 days and 7 days post denervation indicative for an activation of the PERK and IRE1 arm of the UPR. In denervation, also the expression of Grp78/BiP and Chop was also increased (127). These data show that denervation activates the PERK and IRE1 arm of the UPR in skeletal muscle. It also showed that the proapoptotic transcription factor CHOP, also a target gene of the PERK arm of the UPR, is involved in denervation induced muscle atrophy. Specifically, CHOP deficient mice exhibited an increased skeletal muscle wasting in response to denervation (127). Parveen et al., demonstrated an increased mRNA expression of Grp78, Atf4, and Atf6 and an increased protein content of PERK, ATF4, CHOP, IRE1α, and XBP1s in the gastrocnemius of mice in response to 14 days of denervation (128). They also found that a myofiber-specific ablation of Xbp1 attenuated denervation-induced skeletal muscle atrophy in mice (128). A further study in mice revealed that the PERK target gene Sestrin2 (Sesn2) that is a stress responsive protein and protects cells from cell death by maintaining mitochondrial homeostasis (129) is upregulated in the gastrocnemius two weeks after denervation (130). Knockdown of Sesn2 aggravated denervation-induced skeletal muscle atrophy. These data indicate that the PERK arm of the UPR *via* upregulation of Sestrin2 attenuates denervation-induced skeletal muscle atrophy (130). However, because the IRE1/XBP1 arm of the UPR has also been shown to increase Sesn2 expression, this effect is not unique for the PERK arm (131). In summary, these data reveal a complex involvement of the different UPR arms in various atrophy models depending on age and muscle types. Further studies are needed to decipher the involvement of the individual UPR branches in specific atrophy conditions. Generally, it seems that dependent on the muscle atrophy trigger, different branches of the UPR are activated. One further example for this hypothesis is the differential expression of the UPR in sarcopenic patients suffering from either chronic obstructive pulmonary disease (COPD) or lung cancer cachexia. While lung cancer cachexia-patients show an increase in many ER stress markers and all three UPR branches, only few ER stress markers and the IRE1α branch of the UPR are increased in COPD patients (132).

#### The Role of ER Stress and UPR in Sepsis and Inflammation

Almost all major ER stress related genes have been found to be upregulated in sepsis, as summarized by Khan and colleagues (133). Moreover, the UPR is able to regulate cytokine production at various steps, from stimulation of pattern recognition receptors (PRR), to modulation of inflammatory signaling pathways, and the regulation of cytokine transcription factors (134). These reviews (133, 134) based on a significant number of original articles demonstrating that there is a connection between sepsis, inflammation and the UPR pathway. However, if these UPR pathways are also relevant for ICUAW has not been shown.

In sepsis, expression of the pattern recognition receptors Toll-like receptor (TLR) 2 and TLR4 as well as their downstream pathways are induced in skeletal muscle (31). Specifically, we recently showed that the acute phase protein serum amyloid A1 (SAA1) *via* TLR2 and TLR4 activates NF-κB p65, that translocates to the nucleus, binds to NF-κB response elements in the promoter region of its target genes and increases their expression (31). Inhibition of NF-κB inhibited sepsis-induced muscle wasting in mice (31). In addition, TLR2 and TLR4 have been shown to engage IRE1α to promote cytosolic splicing and activation of XBP1 to increase the expression of proinflammatory cytokines, such as IL-6, to regulate innate immune responses in macrophages (135). Our data also revealed that SAA1 *via* TLR2 and TLR4 and the NF-κB pathway increased the expression of IL-6 in myocytes (28, 31). However, if SAA1 binding to TLR2 and TLR4 also affects the IRE1α/XBP1/IL-6 axis in myocytes has not been tested. Moreover, the expression of several TLR as well as their downstream signaling adaptor, myeloid differentiation primary response 88 (MyD88), are increased in response to denervation in skeletal muscle of mice. Importantly, deletion of Myd88 inhibited the activation of XBP1 and attenuated muscle atrophy in response to denervation (128). These data indicate that TLR and TLR signaling are connected to UPR during inflammation.

The skeletal muscle has been shown to be affected by and to react to immunologic conditions such as proinflammatory stimuli (30–32, 136). The involvement of the UPR in the pathogenesis of different myopathies such as Sporadic inclusion body myositis, Myasthenia gravis, Duchenne muscular dystrophy has been reviewed recently (137). In immune cells, ER stress and the UPR are implicated in the regulation of the inflammatory state (138). A couple of studies demonstrated that also in myocytes, proinflammatory stimuli regulate UPR signaling. For example, acute muscle injury by cardiotoxin injections resulted in an inflammatory response and an increased expression of ER stress and UPR genes, including chaperones and genes from all three branches of the UPR. A link between increased cytokines and ER stress in skeletal muscle has also been suggested by the observation that the UPR is disrupted in inflammatory muscle diseases (139).

Interestingly, Interferon gamma (IFN-γ) treatment of myocytes causes a muscular immune response and activates the IRE1α and PERK but not the ATF6 arm of the UPR. Specifically, IFN-γ caused ER stress in mouse myogenic progenitor cell-derived myotubes *in vitro*. In addition, ER stress was also caused by cardiotoxin injections in tibialis anterior *in vivo*. At base line myoblasts and differentiated myotubes do not express class I major histocompatibility complex (MHC) I and MHCII (140). However, when myotubes were treated with IFN-γ the expression of MHCI, TLRs and proinflammatory cytokines (i.e., IL-1β, IL-6) was considerably increased. This response was further triggered by inhibition of ER stress through 4-PBA. The ER stress inducing agents tunicamycin and thapsigargin prevented the IFN-γ-induced expression of MHCII and MHCI molecules. Inhibition of IRE1α attenuated the UPR stressor-mediated reduction of these immunobiological molecules (141). These data indicate that the IRE1α arm mediates immunobiological suppression in myocytes during inflammation. Notably, not only does inflammation affect the UPR but this also works the other way around. IRE1α induces JNK signaling, which regulates many inflammatory genes (68). IRE1α signaling also activates the NF-κB signaling pathway that induces multiple inflammatory cytokines, such as IL-6. In addition it has been demonstrated that IRE1α and IKK are linked by TRAF2 to activate NF-κB (142) and this pathway was also activated in inflammatory skeletal muscle atrophy (30, 31).

There is strong evidence that ER stress induces inflammation by activating inflammasomes (143). Inflammasomes are multiprotein complexes that act as scaffolds for caspase-1-mediated maturation and secretion of inactive pro-IL-1β and pro-IL-18 to active IL-1β and IL-18 (144). Both IL-1β and IL-18 are involved in sepsis (145), sepsis-induced muscle failure (30) and sepsis-induced cardiomyopathy (35). Cytoplasmic receptors of the nucleotide-binding domain (NOD)-like receptor (NLR) family are key inflammasome components (144). The NOD-, leucine-rich repeat (LRR)- and pyrin domain-containing protein-3 (NLRP3) is one of the best-characterized NLR proteins able to form an inflammasome complex and all components of the NLRP3 inflammasome are contained in the skeletal muscle (146). Importantly, ER stress has been reported to activate the NLRP3 inflammasome with a subsequent increase in IL-1β secretion. Because this effect was independent of the UPR a contribution of a novel ER stress response signaling pathway was proposed (143). These findings taken together strongly indicate that there is a direct link between ER stress and inflammation in skeletal muscle and given the role of proinflammatory cytokines in muscle atrophy also a connection between ER stress and ICUAW is very likely. This however needs to be proven.

The role of circulating and tissue residing immune cells for ICUAW in general and ER stress and UPR in particular is not well understood. However, novel technical advances may provide new insights into the role of the various different cell types contained in muscle for the pathogenesis of muscle wasting in critically ill patients. For example, using a combined approach of single-cell RNA sequencing and single-cells mass cytometry, ten different mononuclear cell types (i.e., muscle satellite cells, fibro-adipogenic progenitors, macrophages, neutrophils, endothelial cells, B cells, T cells, glial cells, smooth muscle and mesenchymal cells and scleraxis-positiv tenocytes) were mapped in adult mouse muscle (271). Another study used single-cell RNA-sequencing to classify single cells isolated from tibialis anterior muscle into myocytes, endothelial cells, fibroblasts, mesenchymal stem cells, macrophages, neutrophils, T-cells, B-cells, and dendritic cells. These data indicate that several classes of immune cells are contained in muscle. Importantly, early during sepsis, a decrease in the proportion of most non-immune cell populations and an increase in immune cells (i.e., neutrophils and macrophages) was observed in muscle of male and female mice subjected to the cecal ligation and puncture (CLP) and faecal-induced peritonitis (FIP) models of sepsis. These findings persisted for one month after sepsis (272). In summary, these data implicate that immune cells may be involved in sepsis-induced muscle wasting. However, if UPR in any of these muscle residing cells contributes to ICUAW has not been investigated and is far from being understood. Likewise, if ER stress and UPR affect the levels of immune cells in muscle tissue during acute or chronic injury is also unknown. Nevertheless, these novel techniques will allow us to investigate the function of UPR in any of these cells and its importance for ICUAW.

#### Hypoxemia and Hypercapnia Cause ER Stress and Muscle Wasting

Both hypoxemia and hypercapnia are associated with an increased mortality in critically ill patients, particularly in those patients with acute respiratory distress syndrome (ARDS).

##### Hypoxemia

Hypoxemia induces skeletal muscle atrophy in male Sprague-Dawley rats (273) and male C57BL/6J mice (274), and was implicated to play a role in muscle wasting of patients with chronic obstructive pulmonary disease (275). Hypoxemia-induced muscle wasting was shown to increase MuRF1/Trim63 and atrogin-1/Fbxo32 expression possibly mediating muscle wasting (de Theije et al., 2018). However, hypoxia is also a strong activator of the oxygen sensitive transcription factor hypoxia-inducible factor 1 (HIF-1). Once activated, HIF-1 increases the expression of genes involved in angiogenesis such as vascular endothelial growth factor A (VEGFA) and erythropoiesis as well as glucose and energy metabolism [for Review (276)]. Previously it has been shown that hypoxia and UPR are closely connected with each other. In their study Pereira et al., nicely demonstrated in human neuroblastoma cells that UPR induces VEGF mRNA expression and that ATF4 but not XBP-1s mediated this response (277). They also showed that cooperation of the HIF-1 and UPR pathways led to a greater induction of VEGF expression when compared to activation of either pathway alone (277). It is very likely, especially in critically ill septic patients that pneumonia, edema, and use of vasoconstrictive agents such as norepinephrine hinder oxygen delivery causing hypoxia in peripheral tissues such as skeletal muscle. Hypoxia mediated activation of UPR and HIF-1 may in turn accelerate muscle wasting. However, if the cooperation of both pathways contributes to ICUAW warrants further investigation.

##### Hypercapnia

An elevation of blood carbon dioxide levels called hypercapnia is often observed in critically ill patients with acute respiratory distress syndrome (ARDS) during protective mechanical ventilation (278) and may contribute to ICUAW. It is also frequently observed in patients with chronic obstructive pulmonary disease (COPD). Hypercapnia has been shown to cause skeletal muscle atrophy in human patients and male C57Bl/6 mice as well as cultivated myotubes (279, 280). Hypercapnia-induced muscle wasting was shown to be mediated by a decreased muscular protein synthesis (280) and an increased protein degradation *via* the AMP-activated kinase/FOXO3a/MuRF1 pathway (279). Recent evidence suggests that both acute and chronic hypercapnia impair ER function and cause UPR. A current review eluted on the distinct mechanisms by which hypercapnia and UPR pathways are interconnected in the setting of acute and chronic pulmonary diseases (281). Nevertheless, the importance of these pathways or their interaction for ICUAW is not well understood.

#### Regeneration and Satellite Cells

Many ICU survivors experience a long-lasting impairment of muscle function that may even be apparent five years after their acute illness (44, 45), which is indicative for a perturbed muscle regeneration. This is surprising given the fact that skeletal muscle has a tremendous capacity for regeneration (147). Regeneration of skeletal muscle is realized by muscle residing stem cells, called satellite cells, that are located between the sarcolemma and the basal membrane of myofibers in a quiescent state (147). In response to myofiber damage, satellite cells get activated, proliferate and differentiate into myoblasts that in turn fuse with injured myofibers (147). Although the majority of satellite cells differentiate into the myogenic lineage, few of them undergo self-renewal, return to a quiescent state and replenish the satellite cell pool. Myogenic differentiation requires a well-controlled expression of myogenic transcription factors (148), myoblast-fusion proteins (149, 150) and contractile proteins (151). This involves the sequential expression of the transcription factors paired box gene 3 (Pax3) and Pax7 followed by the expression of myogenic regulatory factors (MRFs) such as Myf5, MyoD, Myogenin, and MRF4. The role of myogenesis in maintaining skeletal muscle size is well described (152); however, its importance in critical illness in general and ICUAW in particular is unexplored. Previously is has been reported that regenerative muscle capacity is impaired in ICU patients (153) and septic mice (154); although, genes involved in skeletal muscle regeneration are upregulated in muscle of survivors of critical illness (155). The process of myogenesis and the underlying mechanisms are investigated *in vitro* by using primary satellite cells and myoblasts, mouse-derived C2C12 myoblasts or rat-derived L6 myoblasts, which form multi-nucleated myotubes in low serum conditions. To investigate myogenesis *in vivo*, intramuscular injections of barium chloride (156) or cardiotoxin, a snake venom toxin that causes myofiber necrosis without effects on satellite cells viability (157), and subsequent time course analyses are used.

Previously it has been reported that the UPR mediates selective apoptosis of differentiation-incompetent myoblasts during skeletal muscle formation (158). In these cells, ER stress activated caspase-12, which in turn activated caspase-9 and caspase-3 to mediate apoptosis. Previously it was shown that exclusively the ATF6 arm of the UPR was increased in those myoblasts that undergo apoptosis in response to induction of myogenic differentiation (159). When ATF6 or caspase-12 were inhibited, myoblast apoptosis was reduced and the formation of multi-nucleated C2C12 myotubes was attenuated. An increase in caspase-12 was also observed in skeletal muscle during embryonic development. These data suggest that the ATF6 arm of the UPR is involved in selective apoptosis of those myoblasts that are susceptible to stress (159). However, caspase-12 deficient mice develop normally (160), indicating that caspase-12–dependent apoptosis is not essential for muscle development. Interestingly, activation of ER stress has been shown to promote myofiber formation (161). When ER stress was activated by tunicamycin or thapsigargin differentiation-associated apoptosis of myoblasts was increased. Nevertheless, the surviving myoblasts were resistant to apoptosis and differentiated well into contracting myotubes. These data suggest that during myogenesis differentiation incompetent myoblasts are specifically targeted by ER stress (161).

The other arms of the UPR are also implicated during myogenesis. A transient activation of the PERK/eIF2α arm of the UPR and its downstream target, CHOP, has been observed in a subset of myoblasts during differentiation (162). Knockdown of CHOP expression lead to an earlier and more robust myogenic differentiation, whereas its overexpression delayed differentiation. Cells that expressed CHOP did not express the myogenic regulatory factors MyoD and Myogenin indicating that CHOP inhibits myogenic differentiation by inhibition of Myod gene expression.

The IRE1 arm of the UPR leads to the generation of XBP1s, a potent transcription factor (163). XBP1 was shown to be a target gene of the transcription factors MyoD and Myogenin (164) that are essential for myogenic differentiation suggesting a connection between XBP1 and myogenesis. XBP1 regulates the expression of genes involved in maintenance of ER function, growth, and DNA damage and repair pathways in myoblasts as well as in myotubes (165). Overexpression of XBP1 inhibited the expression of myogenic differentiation markers, such as Myh7, Myh4 and Mef2c, and led to formation of shorter and less mature C2C12 myotubes during differentiation. This effect was caused by XBP1-mediated induction of Muscle, Intestine and Stomach Expression 1 (Mist1)/Basic Helix-Loop-Helix Family Member A15 (BHLHA15) expression. Specifically, XBP1 binds to the Mist1 promoter in myoblasts, XBP1s and ER stress increase the expression of Mist1 and downregulation of XBP1 reduced the ER stress-induced Mist1 expression (166). Mist1 is a transcription factor that inhibits the activity of MyoD (166). In summary, these data indicate that XBP1 plays a role in myogenic differentiation by induction of Mist1-mediated inhibition of MyoD (165).

The role of satellite cells in general and the UPR in particular in ICUAW is not well defined. Nevertheless, previously it has been shown that the downstream target of the PERK arm of the UPR eIF2α is constitutively phosphorylated in quiescent satellite cells and that this phosphorylation is decreased in activated satellite cells (167). When a phosphorylation resistant mutant of eIF2α was expressed (i.e., eIF2αS51A) or PERK was deleted satellite cell quiescence was lost as indicated by an increased Myf5 and MyoD expression. Satellite cells that express eIF2αS51A are able to undergo terminal differentiation and subsequent fusion with myofibers. However, these satellite cells are incapable to undergo self-renewal. Interestingly, this study also showed that inhibition of eIF2α dephosphorylation using the salubrinal-derivative Sal003 promoted satellite cell self-renewal *ex vivo* (168). Sal003 inhibits the serine/threonine phosphatase GADD34/PP1, which dephosphorylates phosphorylated eIF2α, and therefore keeps eIF2α in a phosphorylated state (169). In addition, inhibition of eIF2α dephosphorylation by Sal003 retained the myogenic capacity of satellite cells when transplanted in skeletal muscle of a mouse model of Duchenne muscular dystrophy *in vivo* (168). However, these data are in contrast to recently published work were eIF2αS51A overexpression was shown to cause cell death and diminish the differentiation of cultured myoblasts (162). Differences in the experimental approaches and techniques as well as the cell types used for analyses might account for these discrepancies (162, 168).

In summary, these data show that ER stress and UPR play a key role in satellite cell function and fate, skeletal muscle regeneration and myogenesis. However, it is currently unknown to which extend satellite cell function and regeneration are involved in ICUAW and even more so if ER stress and UPR are involved in this process.

#### ER Stress Signaling at the Interface Between the ER and Mitochondria

To relive ER stress higher amounts of ATP are needed especially for a proper function of newly synthesized ER chaperone proteins. This suggests a close connection between the ER and mitochondria, which are the main site of ATP production in myocytes. Because muscle is a highly energy consuming tissue the interaction between the ER and mitochondria during ER stress possibly affects its function and adaptation processes. This is supported by the observation that tunicamycin-induced ER stress causes a translocation of mitochondria to the ER. These translocated mitochondria were reported to increase ATP production, which increases the intracellular ATP pool (282). The transport of ATP and signaling molecules between the ER and mitochondria occurs at interfaces between both organelles, which are called mitochondria-associated ER membranes (MAM) (283). This interface is realized by specific proteins localized to the outer mitochondrial membrane and the ER (284). MAM are involved in ER stress, calcium signaling, mitochondrial dynamics, lipid trafficking, initiation of apoptosis, and autophagosome formation [for review (284–286]. Interactions between ER and mitochondria in skeletal muscle and its effects on calcium homeostasis and reactive oxygen species has been reviewed recently (287). MAM have also been shown to influence insulin signaling through different pathways, including those associated with ER stress responses, mitochondrial function, and inflammation. A recent review addressed possible mechanisms underlying MAM-associated insulin resistance (Cheng et al., 2020). In addition, MAM have also been shown to be affected by insulin resistance, obesity, and type 2 diabetes. However, a decrease (Tubbs et al., 2018) as well as an increase (Arruda et al., 2014;Thoudam et al., 2019) of MAM in murine and human skeletal muscle as well as cultivated myocytes has been shown in response to metabolic and ER stress. These conflicting results are possibly due to differences in the techniques used to investigate MAM structure and function in skeletal muscle and myocytes. Likewise, the data on changes of the amount of proteins that mediate tethering of mitochondria to the ER in response to metabolic stress are not uniform. It is therefore difficult to assess the functional impact of MAM modifications for muscle. Also, no data are available for the role of MAM in muscle of critically ill patients, therefore, further analyses are needed to conclude if MAM are involved in the pathogenesis of ICUAW.

#### Physiotherapy and Mobilization

To prevent sequelae of ICU treatment, critically ill patients receive physiotherapy and if applicable early mobilization. The goal is to prevent muscle loss and to maintain and support muscle function, as reviewed elsewhere (41, 170). Protocol-based early mobilization has been reported to improve muscle function and reduce insulin resistance in ICU patients, and is recommended in guidelines (171–174). In general ICU patients, early mobilization has been shown to be clinically beneficial (174–176). If additional physiotherapeutic measures, such as neuromuscular electrical stimulation (NMES), have an effect on muscular structure or function is debated. Some reports described that such interventions prevent muscle atrophy and improve glucose metabolism and physical function, whereas others did not find any effect (102, 177–182). Whole-body vibration (WBV) has been shown to be effective for maintenance of muscle mass and strength in elderly individuals. However, its impact on long-term outcome of ICU patients has not been investigated, although it is feasible in critically ill patients (40, 183, 184). We recently investigated if additional muscle activating measures (e.g., NMES or WBV) have an effect on muscle structure and function when applied in combination with daily protocol-based physiotherapy in critically ill patients (SOFA ≥ 9). We observed that muscle integrity was maintained and found an increased myocyte cross sectional area in the intervention group as compared to those patients that received daily protocol-based physiotherapy only. However, early administration of additional muscle activating measures did not improve muscle strength or function in the critically ill (41). Although early mobilization, physiotherapy, muscle activating measures and physical training are quite distinct in terms of the intended goals and the applied work load, it is noticeable that acute and chronic exercise have been associated with ER stress. For example, a single strong bout of exercise and chronic exercise caused an activation of different branches of the UPR. However, whether or not mobilization therapy increases or decreases ER stress and UPR, if this interferes with the effects of other ICUAW risk factors on ER stress and if this has an effect on muscle structure and function is not known. It is possible, but not proven, that muscle activation measures aggravate UPR that is already induced by immobilization, inflammation, and treatment and that this is time, context, dose and frequency dependent.

If applicable critically ill patients receive a structured physiotherapy during ICU treatment (185). The effects of physiotherapy and mobilization on ER stress-induced UPR with regards to muscle structure and function are uncertain. Although not directly comparable to physiotherapy endurance and resistance exercise have been shown to regulate UPR in muscle. The effects of exercise on UPR were often investigated in rodent models that combined exercise with metabolic stress induced by, among others, type 2 diabetes mellitus and or high-fat diet. In these models, type 2 diabetes mellitus induced by streptozotocin injection and high-fat diet caused glucose intolerance, induced ER stress and endoplasmic reticulum-associated degradation (ERAD) in skeletal muscle of male mice. Swimming (1h per day, 5 days per week for 6 weeks) of these mice improved glucose intolerance and alleviated ER stress in skeletal muscle (186). Specifically, type 2 diabetes mellitus significantly increased the phosphorylation of IRE1α and ATF6 expression in skeletal muscle and swimming alleviated this response. In contrast, the PERK branch of the UPR remained unaffected by type 2 diabetes mellitus and exercise. Similar findings have been reported in muscle biopsy specimens from vastus lateralis from young and old untrained men in response to resistance exercise. Acute resistance exercise led to an increase in both XBP1 and XBP1s, indicating that exercise may stimulate the IRE1α and ATF6 arms of the UPR. In contrast, acute resistance exercise did not affect the PERK pathway as no increase in Atf4, Chop, Gadd34, or eif2a mRNA expression and eIF2α phosphorylation were found 24 hours and 48 hours after exercise (187). In contrast to swimming and resistance exercise, it was shown that endurance exercise, when applied in addition to high-fat diet in mice, further increased the expression of several UPR markers when compared to high-fat diet alone. This response was different between the muscle (i.e., soleus vs. tibialis anterior) and organs (i.e., skeletal muscle, liver, pancreas) investigated (188). Importantly, it has been reported that UPR is absent in the heart in response to short-term exercise in adult male Sprague Dawley rats (189) and male and female mice (190). It is also interesting to note that a single bout of running exercise activates the UPR in some (e.g., quadriceps, gastrocnemius) but no other muscles (e.g., erector spinae) (190) indicating a muscle specific regulation and possibly function of the UPR. Muscle specific (soleus vs. tibialis anterior) and age-related regulations of ER stress markers have also been observed in adult (9 month) and old (29 month) male rats following 14 days of hindlimb unloading by tail-suspension and 13 days of reloading (122). In summary, these data imply that the UPR is differentially regulated in a muscle- and tissue-specific manner that depends on the type of exercise applied (e.g., swimming, resistance- or endurance exercise) and its duration (e.g., short- or long-term, single bout) as well as the preexisting metabolic situation and age. An additional level of complexity is added when the different branches of the UPR are considered. Whether or not physiotherapy and early mobilization are able to increase or decrease UPR, if this adds on to preexisting UPR caused by inflammation, immobilization, insulin resistance, and if this has beneficial or detrimental effects on skeletal muscle in critically ill patients needs further investigation. Given the kinetics of UPR it will be interesting to know if there is a time window where critical illness-induced and therapy related ER stress and UPR interfere with each other and aggravate muscle damage, which most certainly will have clinical implications.

These data show that exercise as well as immobilization are able to activate ER stress and UPR and that UPR is involved in the control of muscle mass and function. However, to which degree immobilization- vs. mobilization-mediated UPR activity affects the occurrence of ICUAW and if this knowledge can be used therapeutically is not known.

#### Pharmacological Targeting of the UPR in ICUAW

Given its importance in multiple cellular processes and human diseases such as cancer, neurodegenerative disease (e.g., Alzheimer’s disease, Parkinson’s disease, amyotrophic lateral sclerosis, Huntington’s disease) and metabolic disorders (e.g., type 2 diabetes mellitus) attempts have been undertaken to pharmacologically target the UPR. These efforts have been summarized recently (191). However, UPR modulators have rarely been tested in critically ill patients or ICUAW, which is not surprising as several critical functions of the UPR in muscle are not fully understood and sometimes controversial. As already mentioned, preclinical animal models revealed that targeting specific UPR branches or mediators may have beneficial or detrimental consequences for disease severity or progression which depends on the disease context. Among others, disease-, tissue- and fiber type-specific activation of distinct UPR pathways that often follow a time course complicate decision making for the therapeutic usage of UPR modulators. Many preclinical disease models are based on otherwise healthy young rodents that do often not have contributing risk factors that may affect the disease course, which is in contrast to the critically ill especially on medical ICUs. These studies are mainly performed in male, some in female rodents but usually not in animals of both sexes. In order to evaluate therapeutic effects especially in muscle results from multiple muscles (e.g., slow- and fast-twitch muscles) need to be obtained, which is often not the case. Finally, although there are many valid reasons to choose a preemptive treatment in situations where the onset of a disease and even its cause is very well defined, this is in great contrast to the clinical reality. These limitations need to be considered when decisions are made to translate from preclinical to clinical applications of UPR modulators.

##### Chaperones

One consequence of UPR activation is an increased expression of chaperone proteins, which will enhance the protein folding capacity of the ER. Therefore, it appears logical to apply chaperones to assist protein folding and to reduce ER stress. The two most commonly used chaperons that are used *in vitro* and *in vivo* in animal models as well as in human patients are the chemical chaperones Tauroursodeoxycholic acid (TUDCA) and 4-phenyl butyric acid (4-PBA). On the basis of cell culture and pre-clinical studies described in the previous chapters, TUDCA and 4-PBA represent promising therapeutic options for those myopathies that are associated with ER stress. Both have previously been shown to be effective in inhibiting the ER stress-mediated lipotoxicity of pancreatic beta-cells (192). For example, 4-PBA enhanced glucose metabolism by increasing hexokinase activity, glucose consumption and Glut4 mRNA expression in L6 myotubes (193). 4-PBA also increased GLUT4 expression and enhanced glucose metabolism in C2C12 myotubes (194). In addition, TUDCA has been shown to improve the blunted glucose uptake in response to tunicamycin in C2C12 myotubes (116).

TUDCA was also found to be effective in preclinical disease models. For example, loss-of-function mutations of the ER-resident phosphatidate phosphatase lipin1 (LPIN1) causes severe muscle injury in children. Skeletal muscle specific Lpin1 knockout mice develop a myopathy (e.g., reduced muscle force, myofiber necrosis, centronucleated fibers, fibrosis, immune cell infiltration) that is linked to an increased UPR and ER fragmentation. When these mice were treated with TUDCA, ER stress was alleviated and the myopathic phenotype was rescued [e.g., increased muscle force, reduction in myofiber necrosis; (195)]. In addition, TUDCA treatment of obese (i.e., leptin-deficient ob/ob mice) mice attenuated high-fat diet induced ER stress and insulin resistance. When applied to inducible muscle-specific Opa1 knockout mice, which show ER stress, systemic inflammation, and muscle atrophy TUDCA treatment reduced ER stress, improved the number of mitochondria and prevented muscle wasting. These data suggest that attenuation of ER stress improves muscle function in a disease model that is characterized by mitochondrial dysfunction (196). In addition, the effects of 4-PBA were investigated in mice with an I4895T mutation in the type I ryanodine receptor (RyR1), which display muscle weakness and atrophy that is associated with an increase in ER stress markers (e.g., GRP94, CHOP and BIP) in skeletal muscle. Chronic treatment of these mice with 4-PBA reduced ER stress and improved muscle strength (197). Finally, in a rat model of severe burn injury, 4-PBA treatment prevented burn-induced ER stress (i.e., ER swelling, expression of ER stress markers) and attenuated skeletal muscle injury and wasting (198).

However, work done by others showed that neither TUDCA nor 4-PBA restored palmitate-induced insulin resistance in C2C12 myotubes (199). Interestingly, when applied to 20 obese subjects (8 men, 12 women) for 4 weeks TUDCA (1,750 mg/day) improved muscular and hepatic insulin sensitivity, but this response was independent of its effects on ER stress since muscular ER stress markers remained unchanged (200). Because ER stress markers are highly activated in skeletal muscle of Lewis lung carcinoma (LLC) and ApcMin/+ (intestinal and mammary tumors) mouse models of cancer cachexia it was hypothesized that inhibition of ER stress could prevent cancer associated muscle wasting. However, 4-PBA treatment not only accelerated the reduction in muscle strength and mass in LLC-tumor bearing mice but also in healthy control animals. 4-PBA also increased the proportion of fast-twitch myofibers in soleus muscle of both control and LLC-bearing mice. 4-PBA also induced atrophy of primary myotubes *in vitro*. These data indicate that ER stress and UPR pathways are essential for maintaining skeletal muscle mass and strength and maybe protective against cancer cachexia (201). Further studies are needed to address the context-dependent and sometimes negative effects that have been observed with TUDCA and 4-PBA.

##### General UPR Modulators

Targeting ER stress-induced UPR may be beneficial for treatment of myopathies, but it is very important to determine which branch of the UPR is involved in which myopathy because inhibiting all three branches may not be safe as it could lead to unexpected side effects. Consequently, targeting specific UPR branches and their downstream regulators may be a better option. The development of UPR modulators, specifically IRE1α RNase inhibitors, IRE1α kinase inhibitors, PERK inhibitors, eIF2α phosphatase inhibitors, eIF2B activators, and ATF6 activators and inhibitors has been reviewed recently (191). Only few of these compounds have been tested in myocytes or muscle pathologies. We have here listed some examples were UPR modulators have been used (**Table 1**).

**Table 1:** Effects of UPR pathway modulators.

UPR pathway modulator	Model	Effect	Ref
PERK inhibitors			
GSK2656157	Palmitate- and tunicamycin-induced ER stress and musclin (a myokine) expression in C2C12 myotubes.	Inhibition of palmitate-induced musclin expression.	(202)
GSK2656157	Myogenic differentiation; primary urethral muscle derived stem cells and rat-derived L6 myoblasts.	Attenuation of myotube formation.	(203)
GSK2606414	Woozy mice carrying a Sil1 mutation recapitulating Marinesco-Sjögren syndrome (i.e., rare, early onset, autosomal recessive multisystem disorder with cerebellar ataxia, cataracts and myopathy).	Reduced ultrastructural skeletal muscle abnormalities; improved motor performance.	(204)
eIF2a phosphatase inhibitors			
Salubrinal	Neonatal rat cardiomyocytes	Protects cardiomyocytes from doxorubicin-induced apoptosis.	(205)
Salubrinal	Receptor expression-enhancing protein 1 (REEP1) knockout mice; Model for hereditary spastic paraplegias that are genetic neurodegenerative disease.	Increased nerve-muscle connections and enhanced motor functions	(206)
Guanabenz	Mouse model of Oculopharyngeal muscular dystrophy related to the polyA-binding protein nuclear 1 (*PABPN1*) gene	Reduced nuclear aggregates, improved muscle force, protected myofibers.	(207)
Inhibitors of P-eIF2α-mediated translational repression			
Trazodone	Human-induced pluripotent stem cell-derived cardiomyocytes	Cardiotoxic effects; prolongation of action potential possibly involved in QT prolongation, arrhythmia, and ventricular tachycardia.	(208)
Dibenzoylmethane	C2C12 and L6 myotubes	Increased phosphorylation of AMPK, elevated GLUT4 expression and translocation, and increased glucose uptake.	(209)

The compound GSK2656157, an inhibitor of the PERK branch of the UPR, has been used in C2C12 cells. Specifically, Gu et al., investigated the effects of ER stress on the expression of musclin (encoded by Ostn, osteocrin), which is a muscle-secreted cytokine (myokine) that is associated with insulin resistance in type 2 diabetes mellitus. Treatment of C2C12 cells with palmitate or tunicamycin caused ER stress and increased the expression of Ostn, which was attenuated by 4-PBA. Importantly, inhibition of the PERK branch of the UPR by GSK2656157 reduced palmitate-induced Ostn expression whereas inhibition of the IRE1 or ATF6 branch of the UPR had no effect. These data indicate that the PERK- but not the IRE1 or the ATF6 branch of the UPR is involved in palmitate-induced Ostn gene expression (202). In summary, it is possible to inhibit specific UPR branches and these inhibitors are useful to perform mechanistic studies in myocytes. If these inhibitors are also useful to prevent or treat ICUAW *in vivo* warrants further investigation.

#### Novel UPR Modulators From Natural Products

Novel UPR modulators were found to be contained in natural products (210), which could serve as a source for novel lead compounds. Such natural UPR modulators provide an innovative and novel strategy to prevent or treat ICUAW. We have here listed a few promising natural compounds, their source and the model systems they were investigated in (**Table 2**). However, for most of these compounds their effects on ER stress have been investigated in heart-failure models *in vivo* or in cardiomyocytes *in vitro*. Although favorable effects of these compounds have been shown in human patients and animal models, further analyses are needed to elucidate if they also inhibit ER stress and UPR pathways in skeletal muscle as part of their mode of action and if this has favorable effects for the critically ill.

**Table 2:** Natural compounds with effects of ER stress and UPR in muscle.

Natural compound	Source	Mechanism of action and protective properties	Ref.
Anisodamine	Tropane alkaloid extracted from the root of *Scopolia tangutica* Maxim.	Anisodamine mediated inhibition of ER stress (e.g., GRP78, CHOP, caspase 3) protected against myocardial injury after cardiac arrest and resuscitation in rats.	(211)
		In a multicenter, open-label trial on adults with septic shock anisodamine (0.1-0.5 mg per kilogram of body weight per hour) had no effect on hospital mortality or ventilator-free days at 28 days.	(212)
		Anisodamine protects skeletal muscle in a rabbit model of ischemia and reperfusion injury.	(213)
		A combination of anisodamine and neostigmine had favorable effects on survival, hemodynamics and muscle damage in an acute lethal crush syndrome in rats and rabbits possibly *via* activation of the α7 nicotinic acetylcholine receptor-dependent JAK2-STAT3 signaling pathway.	(214)
Baicalin	Flavonoid derived from the roots of *Scutellaria baicalensis* Georgi	Baicalin protected neonatal rat cardiomyocytes from tunicamycin-induced ER stress-associated apoptosis *via* downregulation of CHOP.	(215)
		Baicalin treatment inhibited the production of proinflammatory cytokines in macrophages in response to methicillin-resistant Staphylococcus aureus (MRSA). Baicalin also reduced the mortality of MRSA infected mice.	(216)
		Baicalin inhibited the NOD-like receptor (NLR) family, pyrin containing domain 3 (NLRP3) inflammasome in bone marrow-derived macrophages through augmenting protein kinase A signaling. Baicalin treatment significantly improved survival of *Escherichia coli* infected septic mice.	(217)
		Baicalin inhibited H2O2-induced apoptosis of C2C12 myoblasts and reduced skeletal muscle injury caused by intramuscular H2O2-injections in mice *in vivo*.	(218)
		Baicalin (50 mg/day for 3 months) supplementation attenuated lean body mass reduction in cancer patients with involuntary weight loss.	(219)
		Baicalin increased GLUT4 and PGC-1α mRNA and protein expression in rat-derived L6 myocytes.	(220)
Berberine	Isoquinoline-derived alkaloid, isolated from *Rhizoma coptidis*	Berberine (200 mg/kg/day, for 2 weeks) suppressed myocardial ischemia/reperfusion-injury induced ER stress by reducing the phosphorylation of PERK and eIF2α and decreasing the expression of ATF4 and CHOP in hearts of male rats.	(221)
		Berberine reversed high-fat diet-induced muscle mass-loss by reduction of myostatin, and Smad3 and Smad4 expression.	(222)
		Berberine decreased protein synthesis and increased protein degradation in muscles of normal and db/db mice, a model of type 2 diabetes mellitus. Berberine decreased protein synthesis through a reduction in eIF3-f and increased protein degradation *via* induction of Atrogin-1.	(223)
Tetrahydropalmatine	Isolated from Corydalis turtschaninovii	Tetrahydropalmatine inhibited ER stress and inflammasome activation in the liver of high-fat diet-fed golden hamsters possibly *via* inhibition of the TLR4-NF-κB signaling pathway.	(224)
		Tetrahydropalmatine treatment of C2C12 cells led to an increase in MyoD, myogenin and myosin heavy chain protein amounts and facilitated the formation of large multinucleated myotubes possibly through p38MAPK and Akt.	(225)
Quercetin	Flavonoid contained in many fruits, vegetables, leaves, seeds, and grains; capers, red onions, and kale.	Quercetin inhibited ER stress (i.e., GRP78, GADD153) and reversed adverse cardiac remodeling associated with experimental autoimmune myocarditis induced by porcine myosin injections into male Lewis rats.	(226)
		Quercetin reduced obesity-induced skeletal muscle atrophy by inhibiting inflammatory cytokines, cytokine receptors, and proinflammatory signaling pathways.	(227)
		Intake of quercetin prior to sciatic nerve dissection prevented muscle atrophy in mice by targeting and protecting mitochondrial function in skeletal muscle tissue.	(228)
		Intramuscular injection of quercetin prevented tail-suspension-induced muscle atrophy.	(229)
		Quercetin inhibited obesity-induced skeletal muscle atrophy in high-fat diet-fed obese mice. Quercetin also inhibited TNF-induced MuRF1 and atrogin-1 expression.	(230)
		Quercetin increased viability and exerted antiapoptotic effects on dexamethasone-treated C2C12 cells by improving mitochondrial membrane potential and decreasing oxidative stress.	(231)
		Fourteen days of quercetin treatment reduced the severity of muscle weakness caused by eccentric-induced muscle damage in healthy young men.	(232)
Resveratrol	A polyphenol mainly found in red wine, grape seed and grape skin.	Resveratrol suppressed isoproterenol-induced cardiomyocyte hypertrophy and apoptosis by inhibition of ER stress markers (e.g., GRP78, GRP94, CHOP) in neonatal rat cardiomyocytes.	(232)
		In diabetic rats, resveratrol significantly restored cardiac function, reduced cardiomyocyte apoptosis, and ameliorated ER stress *via* PERK/eIF2α, ATF6/CHOP, and IRE1α/JNK-mediated pathways.	(233)
		Resveratrol treatment (200 mg/kg*day for 21 days) prevented an increase in MuRF1 expression and attenuated muscle atrophy in the 5/6-nephrectomy mouse model of chronic kidney disease. Resveratrol also attenuated dexamethasone-induced MuRF1 expression in C2C12 myotubes possibly due to inhibition of NF-κB signaling.	(234)
		Resveratrol supplementation prior to denervation prevented muscle weight loss and muscle fiber atrophy in mice. Resveratrol suppressed the denervation-induced atrogin-1 expression.	(235)
		Resveratrol attenuated TNF-induced atrophy of C2C12 myotubes *via* reactivation of the Akt/mTOR/FoxO1 pathway and inhibition of MuRF1 and atrogin-1 expression.	(236)
		Resveratrol treatment of mice exposed to seven-days of hind-limb immobilization prevented muscle weight- and limb strength-loss, and improved proteolysis and myofiber atrophy in the gastrocnemius muscle.	(237)
Shikonin	Naphthoquinone derived from *Lithospermum erythrorhizon*	Shikonin improved cardiac function, decreased myocardial fibrosis and reduced ER stress (e.g., GRP78, Caspase-3) in a mouse model of isoproterenol-induced heart failure.	(238)
		Shikonin treatment (10 mg/kg/day, i.p., for 4 days) decreased plasma glucose levels and improved insulin-resistance in spontaneously diabetic Goto-Kakizaki rats. Shikonin stimulated the translocation of GLUT4 to the cell membrane and increased glucose uptake in rat-derived L6 myocytes.	(239)
Sulforaphane	Isothiocyanate found in cruciferous vegetables (e.g., cauliflower, broccoli, kale, cabbage, and Brussels sprouts)	Sulforaphane decreased the expression of ER stress markers (e.g., GRP78, CHOP, caspase-12) and improved the viability of neonatal rat cardiomyocytes exposed to hypoxia/reoxygenation injury *in vitro*.	(240)
		Sulforaphane inhibited Dexamethasone-induced C2C12 myotube atrophy *via* activation of Akt/Foxo signaling.	(241)
		Sulforaphane ameliorated serum starvation-induced atrophy of C2C12 myotubes possibly *via* activation of the Nuclear factor erythroid 2-related factor 2-mediated antioxidant effects.	(242)

## Conclusion

Intensive care unit (ICU)-acquired weakness (ICUAW) is a complex clinical syndrome that complicates treatment of the critically ill and may persist for years in survivors. No effective therapy is in place to prevent or treat this devastating syndrome. Several predisposing risk factors have been identified, which allows mechanistical analyses and may pave the road for therapeutic interventions. A dysbalanced protein homeostasis with increased degradation (via UPS and ALP) and reduced synthesis plays a major role in ICUAW. However, ER stress and the UPR that are involved in many physiological and pathological processes in skeletal muscle are also implicated. Specifically, almost all established ICUAW risk factors (e.g., infection, inflammation, sepsis, multiple organ failure, insulin resistance, immobilization) and treatments (e.g., nutrition, physiotherapy/early mobilization) have also been shown to be directly or indirectly associated with ER stress and the UPR. Nevertheless, due to the complexity of multiple UPR pathways and its numerous upstream activators as well as downstream targets the precise function of the UPR in ICUAW is far from being understood. This situation is complicated by the observation that activation of ER stress-induced UPR may have beneficial or deleterious effects, that its activation differs different muscle types, and that it follows a time-course under some conditions. Although ER stress and the UPR are involved in regulation of skeletal muscle mass, it is unknown how they regulate skeletal muscle atrophy and hypertrophy. If ER stress and the UPR within satellite cells are also important for the long-term residuals of ICUAW needs further investigation. Nevertheless, modulation of ER stress and UPR pathways may be useful to treat ICUAW risk factors as well as ICUAW itself. For that, several activators and inhibitors of ER stress are available. However, before these pharmacological compounds can be used, a deeper understanding of the underlying pathomechanisms is required.

## Author Contributions

MK drafted and revised the manuscript, drafted the Figures and the Tables. JF edited and revised the manuscript, edited the Figures and the Tables. All authors contributed to the article and approved the submitted version

## Funding

This study was supported by the Deutsche Forschungsgemeinschaft (FI 965/5-1, FI 965/5-2, FI 965/9-1, FI 965/10-1 (to JF)) and the German Center for Cardiovascular Research, partner site Greifswald [DZHK 81Z5400153 (to JF)].

## Conflict of Interest

The authors declare that the research was conducted in the absence of any commercial or financial relationships that could be construed as a potential conflict of interest.

## Publisher’s Note

All claims expressed in this article are solely those of the authors and do not necessarily represent those of their affiliated organizations, or those of the publisher, the editors and the reviewers. Any product that may be evaluated in this article, or claim that may be made by its manufacturer, is not guaranteed or endorsed by the publisher.
